# Lipocalin 2 contributes to brain iron dysregulation but does not affect cognition, plaque load, and glial activation in the J20 Alzheimer mouse model

**DOI:** 10.1186/s12974-018-1372-5

**Published:** 2018-11-30

**Authors:** Doortje W. Dekens, Petrus J. W. Naudé, Jan N. Keijser, Ate S. Boerema, Peter P. De Deyn, Ulrich L. M. Eisel

**Affiliations:** 1 Department of Neurology and Alzheimer Research Center, University Medical Center Groningen, University of Groningen, Hanzeplein 1, Groningen, 9713 GZ The Netherlands; 2 Department of Molecular Neurobiology, Groningen Institute for Evolutionary Life Sciences (GELIFES), University of Groningen, Nijenborgh 7, Groningen, 9747 AG The Netherlands; 3 Department of Nuclear Medicine and Molecular Imaging, University Medical Center Groningen, University of Groningen, Hanzeplein 1, Groningen, 9713 GZ The Netherlands; 4 Laboratory of Neurochemistry and Behavior, Biobank, Institute Born-Bunge, University of Antwerp, Universiteitsplein 1, 2610 Antwerp, Belgium; 5 University Center of Psychiatry & Interdisciplinary Center of Psychopathology of Emotion Regulation, University Medical Center Groningen, University of Groningen, Hanzeplein 1, Groningen, 9713 GZ The Netherlands

**Keywords:** Alzheimer’s disease, Lipocalin 2, Neutrophil gelatinase-associated lipocalin (NGAL), Neuroinflammation, Astrocytes, Memory, Behavior

## Abstract

**Background:**

Lipocalin 2 (Lcn2) is an acute-phase protein implicated in multiple neurodegenerative conditions. Interestingly, both neuroprotective and neurodegenerative effects have been described for Lcn2. Increased Lcn2 levels were found in human post-mortem Alzheimer (AD) brain tissue, and in vitro studies indicated that Lcn2 aggravates amyloid-β-induced toxicity. However, the role of Lcn2 has not been studied in an in vivo AD model. Therefore, in the current study, the effects of Lcn2 were studied in the J20 mouse model of AD.

**Methods:**

J20 mice and Lcn2-deficient J20 (J20xLcn2 KO) mice were compared at the behavioral and neuropathological level.

**Results:**

J20xLcn2 KO and J20 mice presented equally strong AD-like behavioral changes, cognitive impairment, plaque load, and glial activation. Interestingly, hippocampal iron accumulation was significantly decreased in J20xLcn2 KO mice as compared to J20 mice.

**Conclusions:**

Lcn2 contributes to AD-like brain iron dysregulation, and future research should further explore the importance of Lcn2 in AD.

**Electronic supplementary material:**

The online version of this article (10.1186/s12974-018-1372-5) contains supplementary material, which is available to authorized users.

## Background

Besides aggregation of amyloid-β (Aβ) and tau, it has become clear that Alzheimer’s disease (AD) pathology is also characterized by neuroinflammation and iron dysregulation.

Neuroinflammation has been recognized to play an important role in AD, and involves chronic activation of microglia and astrocytes in brain regions affected by AD pathology [1–3]. While microglia- and astrocyte-mediated immune responses may initially be neuroprotective, chronically activated brain immune cells may lose certain protective functions and acquire toxic properties [2, 4, 5]. For example, chronic production of reactive oxygen species and pro-inflammatory cytokines by activated glia contributes significantly to AD pathology, by activating pro-apoptotic signaling pathways and promoting further aggregation of Aβ [6–10].

Iron dysregulation is also known to play an important part in AD pathology [11]. Iron accumulation occurs in brain regions affected by AD pathology, particularly in plaques and cell types including neurons and microglia [12–15]. Iron accumulation may contribute significantly to AD pathology by promoting Aβ aggregation, enhancing further pro-inflammatory processes, disturbing mitochondrial respiration, stimulating oxidative stress, and inducing ferroptosis [11, 16].

Interestingly, findings suggest that the protein Lipocalin 2 (Lcn2) may be involved in both neuroinflammation and iron regulation, and might contribute to AD pathology. Lcn2, also known as siderocalin, 24p3, uterocalin, and neutrophil gelatinase-associated lipocalin (NGAL), is an acute-phase protein that is rapidly produced and secreted in response to a wide range of inflammatory and pathological stimuli [17, 18]. Lcn2 plays a role in various processes, including the defense against certain bacterial infections by sequestering iron-loaded bacterial siderophores, mammalian iron metabolism, anti- and pro-inflammatory responses, anti- and pro-apoptotic signaling, and cell migration and differentiation [17, 19–21]. Studies showed that Lcn2 protein levels in blood increase with age and mild cognitive impairment, and are increased in human post-mortem brain tissues in different diseases of the central nervous system (CNS), including AD, Parkinson’s disease, and multiple sclerosis [22–27]. Studies in mice and cell culture showed that Lcn2 contributes significantly to neurodegenerative processes, for example by aggravating pro-inflammatory responses, silencing neuroprotective signaling pathways, and sensitizing brain cells to cell death [25, 27–34]. In the context of AD pathology, in vitro experiments showed that astrocytes produce high levels of Lcn2 in response to Aβ, and that Lcn2 renders brain cells more vulnerable to Aβ-induced cell death [27, 31]. Contrastingly, a few studies concerning sepsis, stroke, and multiple sclerosis reported that Lcn2 exerts significant neuroprotective functions, by promoting anti-inflammatory responses and glial pro-recovery phenotypes [35–37].

Whether Lcn2 exerts significant—either neuroprotective or neurodegenerative—effects on AD pathology has not yet been studied in vivo. Therefore, the aim of the present study was to determine the effect of Lcn2 on AD-like pathology in a mouse model of AD. To this end, we compared behavior, memory functioning, and Aβ-associated neuropathology in J20 mice (a transgenic amyloid precursor protein (APP) overexpressing AD mouse model) and Lcn2-deficient J20 (J20xLcn2 KO) mice. Neuropathological investigation included analysis of hippocampal Aβ plaque load, activation of microglia and astrocytes, and iron load.

## Methods

### Mice

Male wild-type (WT), J20, Lcn2 knock-out (KO), and J20xLcn2 KO mice were studied at the behavioral and neuropathological level. Originally, the J20 AD mouse model (overexpressing human APP with the Swedish and Indiana mutations, under a PDGFB promoter [38]) was obtained from the Mutant Mouse Resource and Research Center (MMRRC stock no. 034836-JAX; former JAX stock no. 006293), and lipocalin 2 knock-out (Lcn2 KO) mice were received from the group of Prof. Dr. T. Mak [39] (both lines are on a C57Bl/6 background). Subsequently, mice were bred in our animal facility, and J20 and Lcn2 KO mice were cross-bred to create a J20xLcn2 KO mouse line. J20 and J20xLcn2 KO mice were hemizygous for the J20 transgene, and Lcn2 KO and J20xLcn2 KO mice were homozygous for the Lcn2 knock-out. Genotypes of the mice were determined by polymerase chain reaction (PCR) (Additional file 1: Table S1). Behavioral experiments started when mice were 11.5 months of age. All mouse experiments performed in this study were approved by the animal ethics committee of the University of Groningen (DEC6851A).

### Behavioral experiments

To study different behaviors and cognitive functions, several behavioral tests were performed, in the following order: home-cage activity measurement, open field, novel location recognition, Y-maze spontaneous alternation, elevated-plus maze, and Morris water maze. All tests were conducted by the same experimenter, who was blinded for the mouse genotypes. The arenas used for the behavioral tests (except the Morris water maze) were cleaned with ethanol in between mice. In total, 61 mice were studied, with *n* = 13–17 mice per genotype (WT: *n* = 17, J20: *n* = 13, Lcn2 KO: *n* = 16, J20xLcn2 KO: *n* = 15). Mice were studied in two cohorts of 30–31 mice, balanced for genotype. More details are included in Additional file 1.

### Home-cage activity

Home-cage activity was recorded for 2 weeks for all mice, by placing each mouse cage underneath a passive infrared (PIR) sensor. Movements were aggregated and stored in one-minute bins on a computer.

### Open field

For the open field test, mice were allowed to freely explore a square arena for 5 min. Movements of the mice were tracked with specialized software (EthoVision XT version 11.5, Noldus, The Netherlands). As a measure of locomotor activity, the total distance moved was analyzed. Also, as indications of anxiety-like behavior, the time spent in the center zone of the arena and the latency to first enter the center zone were measured. More details are described in Additional file 1.

### Novel location recognition

The day after the open field, the novel location recognition (NLR) test started. The NLR test is a hippocampus-dependent recognition memory task, based on rodent’s natural exploratory behavior and preference for novelty [40]. The NLR task consisted of a training and test session, 24 h apart. In both the NLR training and test, mice were allowed to freely explore two identical objects (placed in a square arena) for 10 min. However, in the NLR test, one of the two objects was placed at its original (training) location while the other object was relocated. Object exploration was analyzed, and as a measure of recognition memory, the discrimination index was calculated: (*time spent exploring the* (*to be*) *relocated object*/*total exploration time*)*100. A discrimination index of above 50% indicated a preference for the relocated object. Please find more details in Additional file 1.

### Y-maze spontaneous alternation

As an indication of spatial working memory, mice were studied in the Y-maze spontaneous alternation task [41]. Mice were placed in the center of a white Y-shaped maze, and allowed to freely explore all three arms for 10 min. Trials were recorded and scored for arm entries (by a blinded observer), after which the percentage of spontaneous alternation was calculated. Also see Additional file 1.

### Elevated-plus maze

To further assess anxiety-like behavior, mice were tested in the elevated-plus maze (EPM) [42]. Mice that are more anxious are likely to be more reluctant to explore open and elevated spaces. Mice were placed in the center of the EPM and were allowed to freely explore the maze for 8 min. The crossings into open and closed arms, and the time spent in the open, closed, and center zones were scored. Also see Additional file 1.

### Morris water maze (hidden-platform)

The Morris water maze (MWM) was performed to assess hippocampus-dependent spatial learning and memory [43]. The MWM was performed as described by Van Dam et al. [44, 45], with some modifications. Briefly, in the training phase, mice were trained to learn the presence and location of a platform hidden in the MWM pool. The training phase comprised of eight consecutive days, with one training block (consisting of four trials, each of maximally 120 s) per mouse per day. Swimming trajectories (including escape latency and swimming distance) of the mice were tracked with EthoVision XT software (Noldus, The Netherlands). After eight MWM training days, two probe trials followed, performed 24 h and 48 h after the last training. For the probe trial, the platform was removed from the maze, and mice were allowed to swim freely for 100 s. As a measure of spatial accuracy, the number of crossings through the original platform position was scored, as well as the time spent in the target quadrant and the other quadrants. Please find more details in Additional file 1.

### Immunohistochemistry for detection of Aβ, GFAP, Iba1, and Lcn2

A week after the last behavioral test was finished, mice were terminally anesthetized by intraperitoneal injection with sodium pentobarbital and transcardially perfused with saline and 4% paraformaldehyde (PFA). Brains were then post-fixed in PFA for an additional 24 h, dehydrated, frozen, and cut in coronal sections of 20-μm-thick on a cryostat. Hippocampal free-floating sections were stained for Aβ, glial fibrillary acidic protein (GFAP) and ionized calcium binding adaptor molecule 1 (Iba1), and immunofluorescent co-stainings were performed for Lcn2 and Aβ, GFAP, Iba1, and NeuN. Details of immunohistochemical stainings are included in Additional file 1.

### Histochemistry for detection of iron

For detection of (mostly ferric Fe^3+^) iron, the 3,3′-diaminobenzidine (DAB)-enhanced Perls’ iron stain was used. A staining protocol comparable to that reported by Chen et al. 2015 [46] was used, with some modifications, as described in Additional file 1.

### Quantification of (immuno)histochemical stainings

For the Aβ, GFAP, and iron stainings, the coverage as well as the optical density of the staining was quantified. For analysis of the Iba1 staining, we were interested in obtaining a measure of microglial activation. To this end, the ratio between the cell body size and the total cell size of microglia was analyzed, as described previously by Hovens et al. [47, 48]. For all stainings, quantification was mostly focused on the hippocampus. The Aβ staining was analyzed for the entire hippocampus. The analysis of the GFAP, Iba1, and iron stainings was performed for specific hippocampal sub-regions, including sub-regions of the cornu ammonis sector 1 (CA1) region and dentate gyrus (DG) (as indicated in the results and in Additional file 1: Figure S1). For all stainings, 3–6 hippocampi were analyzed per mouse. More details are described in Additional file 1.

### Statistical analysis

Results were tested by one-way ANOVA followed by Tukey’s multiple comparisons post-hoc test, or by Kruskal-Wallis test followed by Dunn’s multiple comparisons test when data was not normally distributed or variances were unequal. Two-way repeated measures ANOVA was used to analyze the MWM training phase. Statistical analysis was performed using GraphPad Prism version 5.0 for Windows. Data are presented as mean ± standard error of the mean (SEM). Differences between Lcn2 KO mice and J20/J20xLcn2 KO mice were not indicated, since comparisons between these groups were not considered relevant to our study aims. Differences were considered statistically significant when *p* < 0.05.

## Results

### J20 and J20xLcn2 KO mice are equally hyperactive

Home-cage activity of WT, J20, Lcn2 KO, and J20xLcn2 KO mice (11.5 months of age) was recorded for 2 weeks. Home-cage activity measurement revealed clear hyperactivity in J20 and J20xLcn2 KO mice (*p* < 0.0001 and *p* < 0.05 respectively, compared to WT, Additional file 1: Figure S2A, B), corresponding to hyperactive behavior in the open field (*p* < 0.05 compared to WT, Additional file 1: Figure S2C) and in other tests (Additional file 1: Figure. S3). Although the home-cage activity results seem to suggest that J20xLcn2 KO mice might be less hyperactive as J20 mice, this difference was not statistically significant. Thus, it appears that J20 and J20xLcn2 KO mice show clear and equally strong hyperactivity.

Another important basic behavioral characteristic, anxiety-like behavior, was studied in the open field and elevated-plus maze (EPM). Despite an increased time spent in the center zone of the open field in Lcn2 KO mice compared to WT mice (*p* < 0.05, Additional file 1: Figure S4A), no differences were found in the latency to enter the center zone of the open field (Additional file 1: Figure S4B), in the time spent on the open or closed arms in the EPM (Additional file 1: Figure S4C, D) or in the number of EPM arm entries (data not shown). Thus, overall, there seem to be no significant differences in anxiety-like behavior between the studied genotypes.

### J20 and J20xLcn2 KO mice show equally severe memory impairment

**Fig. 1:**
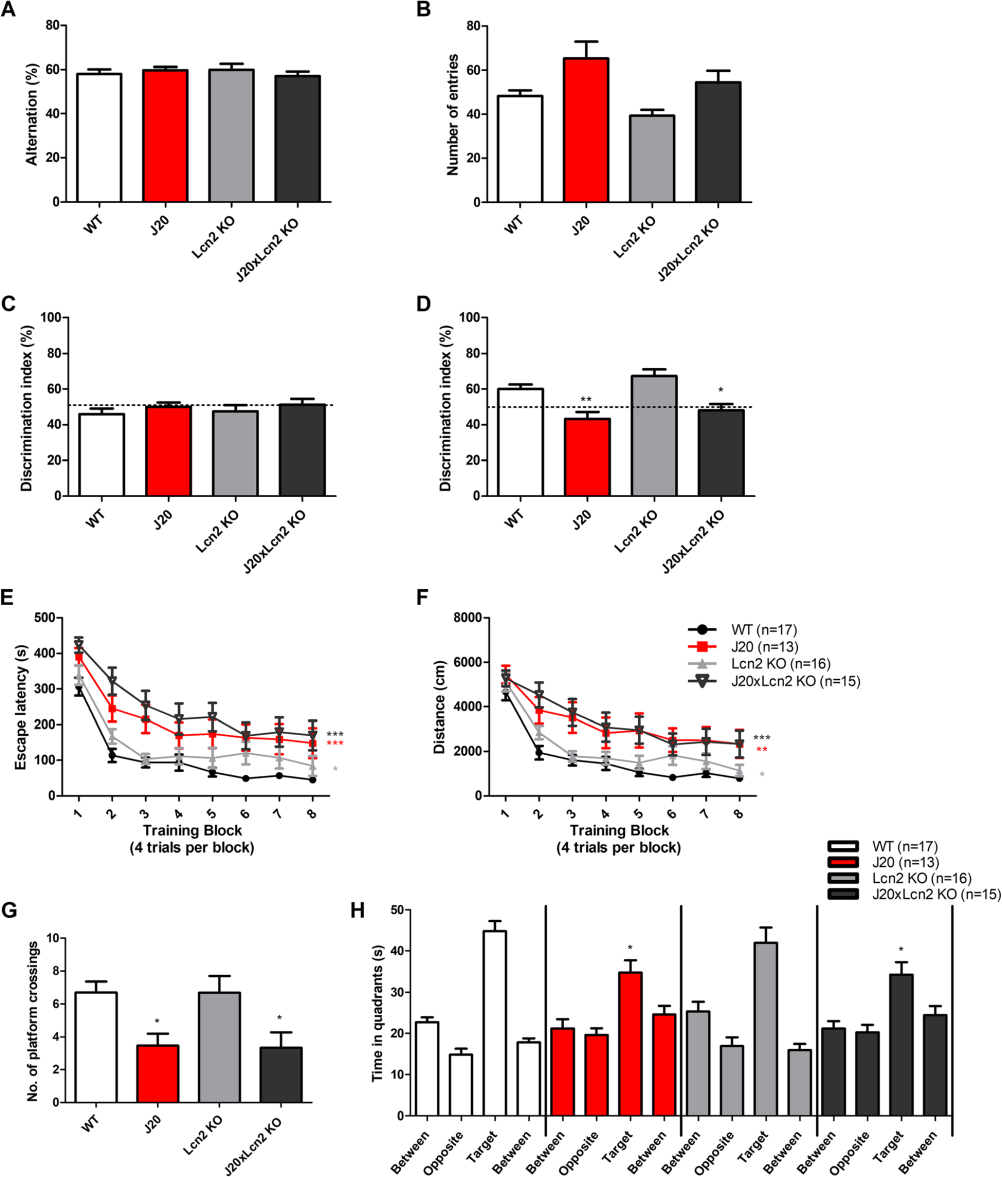
Impaired hippocampus-dependent long-term memory in J20 and J20xLcn2 KO mice, no differences in working memory. **a** Alternation (%) in the Y-maze spontaneous alternation test. **b** Number of arm entries in the Y-maze spontaneous alternation test (10 min). Tested with one-way ANOVA with Tukey’s multiple comparisons test. *n* = 13–17 mice per group (WT *n* = 17, J20 *n* = 13, Lcn2 KO *n* = 16, J20xLcn2 KO *n* = 15). **c** Discrimination index (%) in the novel location recognition (NLR) training and **d** in the NLR test, that was performed 24 h after the NLR training. Dashed line indicates 50% chance level. Of note, one Lcn2 KO and one J20xLcn2 KO mouse had to be excluded from the analysis due to lack of exploratory behavior. *n* = 13–17 mice per group (WT *n* = 17, J20 *n* = 13, Lcn2 KO *n* = 15, J20xLcn2 KO *n* = 14). Tested with one-way ANOVA with Tukey’s multiple comparisons test. **e** Escape latency (s) and (**f**) swim distance (cm) in the training phase of the Morris water maze (MWM). *n* = 13–17 mice per group (WT *n* = 17, J20 *n* = 13, Lcn2 KO *n* = 16, J20xLcn2 KO *n* = 15). Tested with two-way repeated measures ANOVA. **g** Number of platform crossings and **h** time spent in the pool quadrants during the probe trial (24 h after the last training trial). *n* = 13–17 mice per group (WT *n* = 17, J20 *n* = 13, Lcn2 KO *n* = 16, J20xLcn2 KO *n* = 15). Tested with one-way ANOVA with Tukey’s multiple comparisons test. *p < 0.05; **p < 0.01; ***p < 0.0001 compared to WT. No significant differences were present between J20 and J20xLcn2 KO mice

Next, we aimed to assess whether the lack of Lcn2 in J20xLcn2 KO mice caused a difference in working memory compared to J20 mice in the Y-maze spontaneous alternation task. As shown in Fig. 1a, no differences in working memory were found between the studied genotypes. Also, no significant differences were found for the number of arm entries in the Y-maze spontaneous alternation (Fig. 1b).

Subsequently, hippocampus-dependent memory functioning was investigated in the novel location recognition (NLR) task and the hidden-platform Morris water maze (MWM). In the training of the NLR, as expected, none of the groups showed a preference for one of the two identical objects that had been placed in the open field (Fig. 1c, showing a discrimination index of around 50%). In the NLR test (24 h after the training), both WT and Lcn2 KO mice spent more time exploring the relocated object, indicating intact recognition memory (discrimination indices were significantly above chance level (50%), *p* < 0.05 and *p* < 0.01, respectively, Fig. 1d). Lcn2 KO mice did not differ from WT mice in recognition memory. J20 and J20xLcn2 KO mice performed significantly worse compared to WT mice (*p* < 0.01 and *p* < 0.05, respectively) and showed a discrimination index that was not significantly different from chance level, indicating impaired recognition memory. No significant difference between J20 and J20xLcn2 KO mice was observed.

In the MWM J20 and J20xLcn2 KO mice presented impaired learning compared to WT mice, as seen from the reduced slope of the learning curves of the escape latency and swim distance parameters (*p* < 0.01 and *p* < 0.0001 respectively, Fig. 1e, f). Also in the probe trials, performed 24 h and 48 h after the last MWM training, J20 and J20xLcn2 KO had significantly worse memory of the platform position and the target quadrant (Fig. 1g, h, and Additional file 1: Figure S5), as seen from fewer crossings over the position where the platform used to be, and less time spent in the target quadrant (*p* < 0.05).

Thus, J20 and J20xLcn2 KO mice showed equally severe memory impairment in both the NLR test and the MWM. Of note, Lcn2 KO mice showed significantly slower MWM learning curves compared to WT mice, possibly indicating mild memory impairment during the training phase (*p* < 0.05, Fig. 1e, f). However, Lcn2 KO mice performed similar to WT mice in the first and second probe trial (Fig. 1g, h and, Additional file 1: Figure S5).

### J20 mice show increased hippocampal Lcn2 levels in astrocytes

**Fig. 2:**
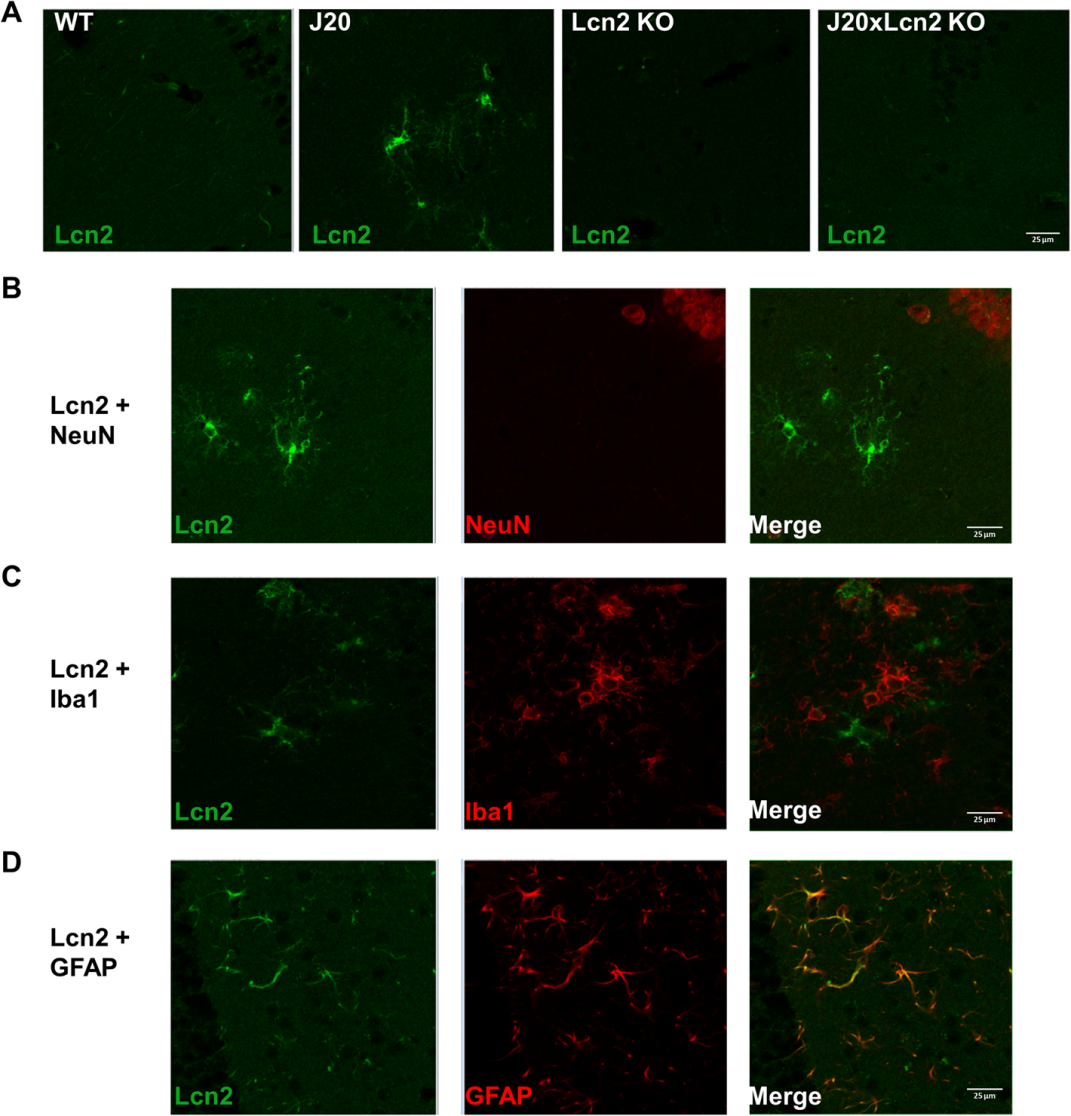
Lcn2 production is increased in the J20 brain, and is localized in GFAP-positive astrocytes. **a** Representative images of fluorescent staining for Lcn2 in the hippocampus of WT, J20, Lcn2 KO, and J20xLcn2 KO mice. **b** Representative images of fluorescent co-staining for Lcn2 and the neuronal marker NeuN, in the J20 hippocampus. **c** Representative images of fluorescent co-staining for Lcn2 and the microglia marker Iba1, in the J20 hippocampus. **d** Representative images of fluorescent co-staining for Lcn2 and the astrocyte marker GFAP, in the J20 hippocampus. Images were acquired with a 63x (oil) objective lens. Scale bar: 25 μm

To confirm that Lcn2 levels are increased in the brains of J20 mice, and to assess the cell type to which Lcn2 was mainly localized, fluorescent stainings were performed. As shown in Fig. 2a, clear Lcn2-positive cells are indeed present in the J20 mouse brain, while WT mouse brains only show faint Lcn2-positive staining. To assess the brain cell type in J20 mouse brains in which Lcn2 was localized, co-stainings were performed for Lcn2 and NeuN, Iba1, and GFAP (Fig. 2b–d). Lcn2-positive staining did not co-localize with the neuronal marker NeuN or the microglia marker Iba1, but did clearly co-localize with GFAP-positive astrocytes.

### J20 and J20xLcn2 KO mice develop similar hippocampal plaque load, and show equal microglia and astrocyte activation

**Fig. 3:**
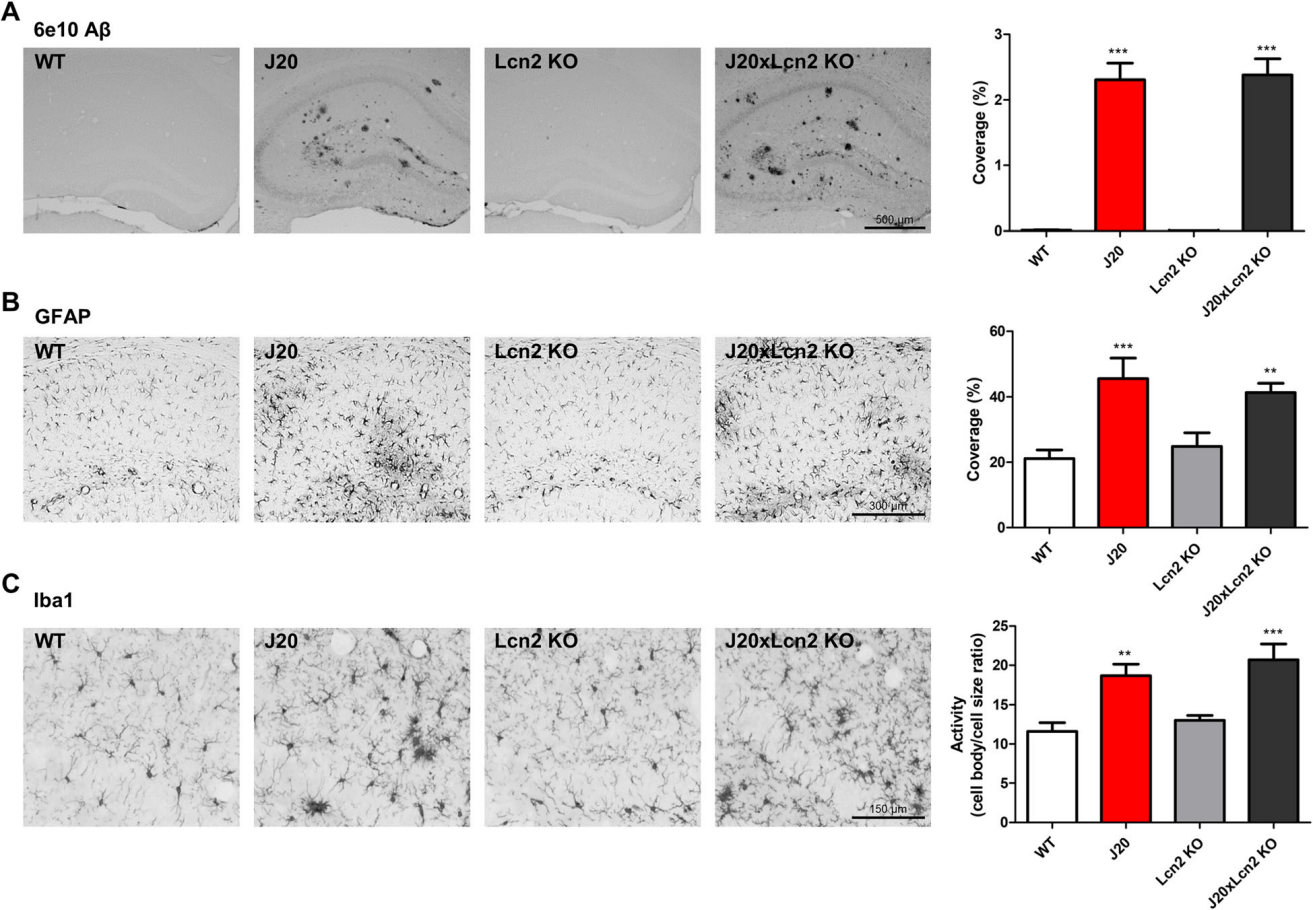
Clear plaque formation and activation of astrocytes and microglia in J20 and J20xLcn2 KO mice. **a** Representative examples of immunohistochemical staining for Aβ with 6e10 antibody (× 50 magnification, scale bar: 500 μm), and (in the outer right column) quantification of the coverage of total hippocampal Aβ staining. **b** Representative examples of immunohistochemical staining for GFAP (× 100 magnification, scale bar: 300 μm), and (in the outer right column) quantification of the coverage of GFAP staining in the dentate gyrus and CA1 region of the hippocampus (combined). **c** Representative examples of immunohistochemical staining for Iba1 (× 200 magnification, scale bar: 150 μm), and (in the outer right column) quantification of microglial activity in the dentate gyrus of the hippocampus. *n* = 8–10 mice per group (WT *n* = 10, J20 *n* = 8, Lcn2 KO *n* = 9, J20xLcn2 KO *n* = 9), 3–6 hippocampi were analyzed per mouse. Tested with one-way ANOVA with Tukey’s multiple comparisons test. **p < 0.01 and ***p < 0.0001 compared to WT. No significant differences were present between J20 and J20xLcn2 KO mice

Although no differences were found between J20 and J20xLcn2 KO mice in behavioral and memory phenotype, it is possible that differences may be present at the neuropathological level. To investigate this, hippocampal slices were stained for (1) Aβ to assess potential differences in Aβ accumulation, (2) GFAP to assess possible differences in astrocyte activity, (3) Iba1 to determine possible differences in microglia activation, and (4) iron to investigate potential differences in iron accumulation. As shown in Fig. 3a, clear Aβ aggregation and plaque load was present in the hippocampus of J20 mice, quantified by measuring the total coverage of Aβ-staining (*p* < 0.0001 compared to WT). J20xLcn2 KO mice, however, showed a similar hippocampal coverage of Aβ-positive staining. Clear astrogliosis (*p* < 0.0001 increase in GFAP coverage compared to WT) and microglia activation (*p* < 0.01 increase in microglia activity compared to WT) were also found in the hippocampus of J20 mice, yet, equal glial activation was found in J20xLcn2 KO mice (Fig. 3b, c). Astrocyte and microglia activation were assessed in specific hippocampal sub-regions. Interestingly, astrocyte activation in J20 and J20xLcn2 KO mice appeared to be induced equally strongly in all hippocampal sub-regions analyzed, as well as the corpus callosum (Additional file 1: Figure S6). In contrast, it appeared that microglia activation may depend more on the specific hippocampal region: while clear microglia activation is found in the dentate gyrus, microglia activation is not significantly increased in the CA1 region (Additional file 1: Figure S7).

### Iron dysregulation is present in the hippocampus of J20 mice, and is less severe in J20xLcn2 KO mice

**Fig. 4:**
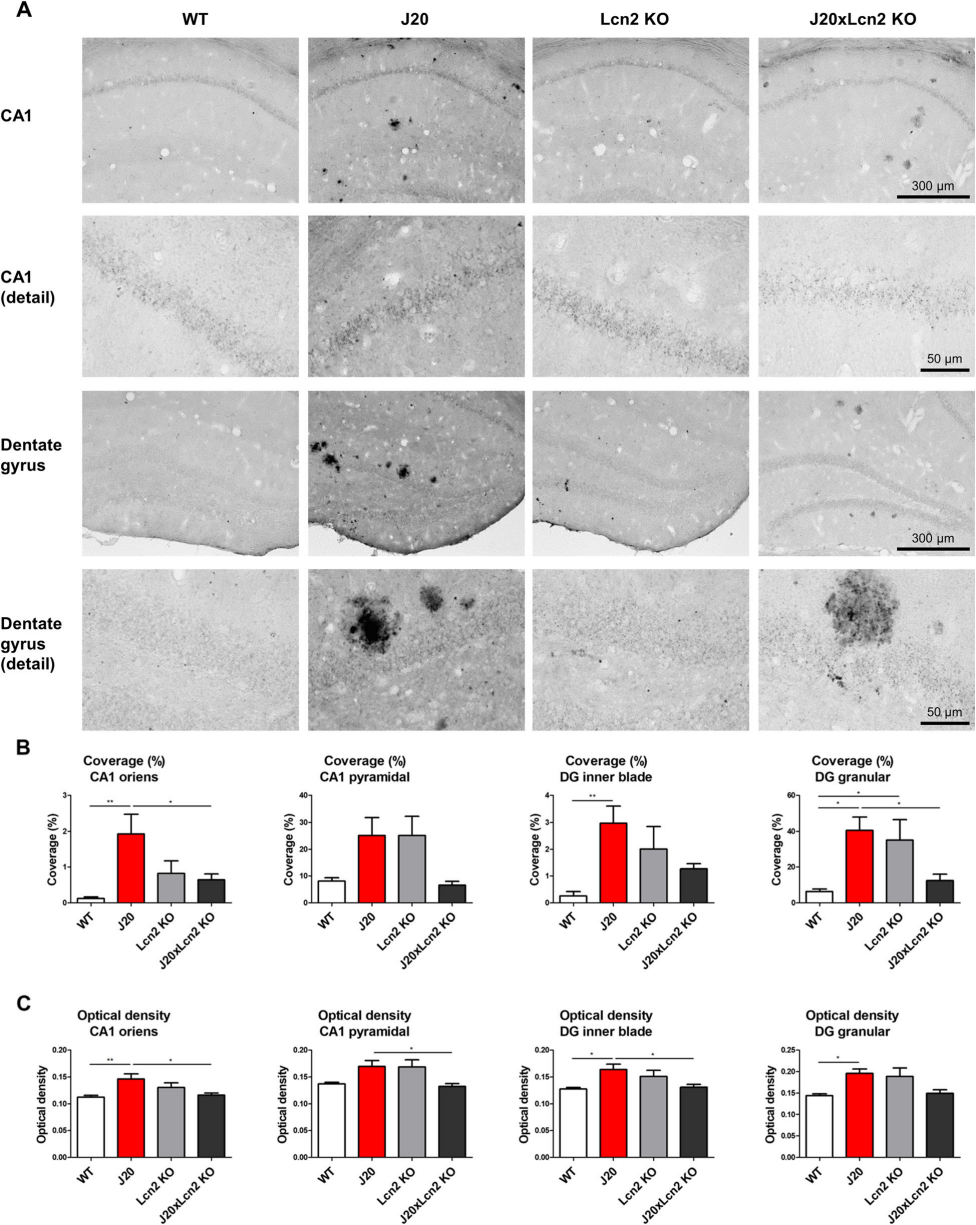
Increased hippocampal iron accumulation in J20 mice, and less severe iron accumulation in J20xLcn2 KO mice. **a** Representative examples of histochemical staining for iron by enhanced Perls’ reaction. Images were acquired and analysis was performed at × 100 magnification (scale bar: 300 μm). Detailed images were acquired at × 400 magnification (scale bar: 50 μm). Examples include a focus on the CA1 and the dentate gyrus regions of the hippocampus. **b** Quantification of the coverage of iron-positive staining, in the stratum oriens and pyramidal layer of the CA1 region, and in the inner blade and granular layer of the dentate gyrus. Of note, due to regional differences in intensity of the iron staining, the iron staining in the CA1 pyramidal and DG granular cell layers was analyzed with a different detection threshold than the detection threshold used to analyze the other hippocampal sub-regions. This explains the higher relative coverages of iron staining found for the CA1 pyramidal and DG granular cell layers, as compared to other hippocampal sub-regions. **c** Quantification of the optical density of iron-positive staining, in the stratum oriens and pyramidal layer of the CA1 region, and in the inner blade and granular layer of the dentate gyrus. *n* = 8–9 mice per group (WT *n* = 9, J20 *n* = 8, Lcn2 KO *n* = 9, J20xLcn2 KO *n* = 9), 3–4 hippocampi were analyzed per mouse. Tested with Kruskal-Wallis test with Dunn’s multiple comparisons post-hoc test. *p < 0.05 and **p < 0.01

Finally, potential differences in hippocampal iron regulation were investigated between genotypes. To this end, hippocampal sections were stained by DAB-enhanced Perls’ staining (detecting mostly ferric Fe^3+^ iron), and the coverage and optical density of iron staining were analyzed in different hippocampal sub-regions. As shown in Fig. 4a, a clear increase in iron staining was detected in the hippocampus of J20 mice, including both plaque-like iron staining and more diffuse and intracellular iron staining (such as the staining seen in the pyramidal and granular neuronal layers in the CA1 and dentate gyrus). As shown in Fig. 4b, c and Additional file 1: Figure S8A, B, quantification of the coverage and optical density of the iron-positive staining confirmed this observation of increased iron staining in J20 mice as compared to WT mice, in many of the studied hippocampal sub-regions (*p* < 0.05 or *p* < 0.01 in different hippocampal sub-regions). Intriguingly, less iron staining is visible in the hippocampus of J20xLcn2 KO mice, as compared to J20 mice (*p* < 0.05 in different hippocampal sub-regions). Moreover, it appeared that Lcn2 KO mice may display stronger hippocampal iron staining as compared to WT mice, in different hippocampal sub-regions.

## Discussion

We report that J20 and Lcn2-deficient J20 (J20xLcn2 KO) mice at 12 months of age show equally severe AD-like behavioral changes, cognitive impairment, plaque formation, and glial activation. Intriguingly, J20xLcn2 KO mice presented significantly decreased AD-like iron accumulation in the hippocampus, as compared to J20 mice.

The reduced hippocampal iron load in J20xLcn2 KO versus J20 mice indicates that Lcn2 contributes to AD-like iron accumulation in the brain. This result corresponds with the finding of Lcn2-mediated brain iron accumulation in an animal model of hemorrhagic stroke [34]. Also, Lcn2 was reported to stimulate ferritin mRNA expression in Aβ-treated cultured astrocytes, indicating a Lcn2-mediated increase in iron storage upon Aβ-challenge [31].

Lcn2 is known to be involved in iron metabolism by binding to bacterial and mammalian siderophores (small iron-binding molecules) present in the body [49–51]. Lcn2 is able to mediate both import and export of iron into/from cells [52, 53]. Under inflammatory conditions, Lcn2 contributes to an iron-retentive response known as the anemia of inflammation, by promoting iron retention in macrophages [54, 55]. Possibly a similar iron-retentive response occurs in the inflamed brain. Indeed, astrocytes, neurons, and especially microglia (the macrophages of the brain) were found to accumulate high levels of non-transferrin bound iron upon inflammatory stimulation [15, 56–61], which might possibly in part be regulated by Lcn2.

This possibility may explain the observed Lcn2-mediated iron retention in J20 versus J20xLcn2 KO brains, and warrants further investigation of involved cell types and siderophores. Moreover, the pathophysiological relevance of Lcn2-mediated brain iron accumulation in AD requires further research. Namely, despite the well-known important role of iron dysregulation in different AD-related pathophysiological processes, the alleviated iron accumulation in J20xLcn2 KO mice as compared to J20 mice is not accompanied by significant changes in other AD characteristics, such as behavior, cognition, Aβ aggregation, and glial activation. It may be possible that the hippocampal iron accumulation in 12-month-old J20 mice is not severe enough (yet) to overwhelm the stable iron storage facilities in the brain, and/or that other compensatory mechanisms induced upon iron dysregulation are (still) sufficient to protect the brain from iron toxicity. This scenario would explain why the difference in iron load between J20 and J20xLcn2 KO mice does not result in significant differences in other AD characteristics. It would be of great interest to study this hypothesis, including the possibility that (Lcn2-mediated) iron dysregulation may become more severe in more advanced AD mice. In these future studies, it would be interesting to for example assess potential differences in levels of labile reactive iron, oxidative stress, and ferroptosis.

Interestingly, the results suggests that Lcn2 may also affect iron homeostasis under healthy unchallenged conditions, as indicated by the increased hippocampal iron levels in Lcn2 KO mice as compared to WT mice. It should be noted that significance between WT and Lcn2 KO mice was often not reached when all four genotypes were compared, while significant differences were found when only WT and Lcn2 KO mice were compared. This finding corresponds with previous reports, in which increased iron levels were found in macrophages and neural stem cells in healthy Lcn2 KO mice [62–64].

Taken together, basal physiological levels of Lcn2 under healthy conditions may be required to maintain iron homeostasis (with absence of Lcn2 leading to iron accumulation in certain cell types, possibly by reduced iron export), while increased Lcn2 levels under pathological conditions may contribute to iron dysregulation and accumulation as well (with increased Lcn2 levels leading to iron accumulation in plaques and certain cell types, possibly by increased iron import). Importantly, although iron homeostasis is tightly regulated, it thus appears that Lcn2 has an important function in iron regulation and distribution in the brain.

No differences were detected in behavior, cognition, plaque load, and glial activation between J20 and J20xLcn2 KO mice. The absence of significant detrimental or beneficial effects of Lcn2 on these AD-like characteristics is notable, taking into account the strong neurodegenerative [22, 25, 27–29, 31–34, 65–69] and neuroprotective [35–37] effects that were reported for Lcn2 in various in vitro and animal models of brain injury and disease. It is possible that Lcn2 indeed does not affect cognition and glial activation in AD, which would provide interesting contrasting information on the role of Lcn2 in neurodegeneration. Alternatively, it is possible that Lcn2 might affect AD pathology in other models of AD. For example, it would be important to validate if Lcn2 might affect cognitive function and pathology in AD models that present more severe neuronal loss (for example APP/PS1KI mice [70]), or in mice at other ages. Furthermore, although clear Lcn2-positive astrocytes were detected, it should be determined whether Lcn2 in the J20 mouse brain reaches concentrations that are comparable to the Lcn2 levels in the human AD brain. Related to this, it would be of interest to investigate AD models which overexpress Lcn2 (besides Lcn2-deficient AD models). Of note, most studies regarding the role of Lcn2 in CNS disease/injury until now were performed in acute models of CNS injury (which may cause stronger, short-term, induction of Lcn2), in contrast to the chronic transgenic AD model studied here (in which—in comparison to models of acute injury—Lcn2 expression may be less strong, which may also be the case for the expression of other inflammatory mediators such as interleukin 1 beta (IL-1β, also see, Additional file 1: Figure S9)). Finally, it should be taken into account that J20 mice overexpress human Aβ while expressing murine Lcn2 (62% identical to human Lcn2 on the amino acid level [71]), which might impact their potential direct or indirect interactions and the translational value to the human disease.

Taken together, more insight into the protective and toxic effects of Lcn2 is required to understand its importance in AD, and its potential as a therapeutic target. Of note, while Lcn2 KO mice are of great value in the study of Lcn2, it should be considered that potential mild developmental disturbances may be present in these mice. As shown in the current and previous studies [62, 63], healthy unchallenged Lcn2 KO mice may display disturbed iron regulation in the periphery and the brain, which might affect important physiological processes such as synaptic plasticity and neurogenesis [62, 72, 73]. Possibly related to this, Lcn2 KO mice appear to present a mild cognitive impairment, as seen from the MWM learning curves in Fig. 3e, f (Additional file 1: Figure S10) and previous reports [62, 72]. Interestingly, this mild cognitive impairment might in part be overcome with age (Additional file 1: Figure S10). To circumvent potential mild developmental disturbances in Lcn2 KO mice, it would be of interest to investigate conditional Lcn2 KO (or conditional Lcn2-overexpressing) mice.

## Conclusions

The results from this study show that Lcn2 contributes to hippocampal iron accumulation in the J20 AD mouse model. As such, this study provides new insights into Lcn2 as a potential functional element in iron dysregulation in the AD brain. Lcn2 did not significantly affect other AD-like characteristics (including behavioral changes, cognitive impairment, plaque load, and glial activation) in the J20 mouse model at 12 months of age. However, it is possible that such effects might surface in other AD models and/or at other ages, which should be investigated in future work.

## Additional file

Additional file 1: Lipocalin 2 contributes to brain iron dysregulation but does not affect cognition, plaque load and glial activation in the J20 Alzheimer mouse model. Contains supplementary information and supplementary figures, belonging to the study ‘Lipocalin 2 contributes to brain iron dysregulation but does not affect cognition, plaque load and glial activation in the J20 Alzheimer mouse model’. (PDF 1866 kb)

## Abbreviations

AD: Alzheimer’s disease
APP: Amyloid precursor protein
Aβ: Amyloid-β
CA1: Cornu ammonis sector 1
CNS: Central nervous system
DAB: 3,3′-Diaminobenzidine
DG: Dentate gyrus
EPM: Elevated-plus maze
Fe^3+^: Ferric iron
GFAP: Glial fibrillary acidic protein
Iba1: Ionized calcium binding adaptor molecule 1
Lcn2 KO: Lipocalin 2 knock-out
Lcn2: Lipocalin 2
MWM: Morris water maze
NGAL: Neutrophil gelatinase-associated lipocalin
NLR: Novel location recognition
PCR: Polymerase chain reaction
WT: Wild type
