# Supplementary material for: Lipocalin 2 contributes to brain iron dysregulation but does not affect cognition, plaque load, and glial activation in the J20 Alzheimer mouse model
Source: J Neuroinflammation. 2018 Nov 30;15:330. doi: 10.1186/s12974-018-1372-5 (PMC6267886; doi:10.1186/s12974-018-1372-5)
Supplement: Supplementary file 1 — Lipocalin 2 contributes to brain iron dysregulation but does not affect cognition, plaque load and glial activation in the J20 Alzheimer mouse model. Contains supplementary information and supplementary figures, belonging to the study ‘Lipocalin 2 contributes to brain iron dysregulation but does not affect cognition, plaque load and glial activation in the J20 Alzheimer mouse model’. (PDF 1866 kb) [file 12974_2018_1372_MOESM1_ESM.pdf]

Additional file 1

Supplementary information and supplementary figures

Journal of Neuroinflammation

Lipocalin 2 contributes to brain iron dysregulation but does not affect cognition, plaque load and glial activation in the J20 Alzheimer mouse model

Doortje W. Dekens, Petrus J.W. Naudé, Jan N. Keijser, Ate S. Boerema, Peter P. De Deyn, Ulrich L.M. Eisel*.

*Corresponding author: Prof. Dr. Ulrich L.M. Eisel. Department of Molecular Neurobiology, Groningen Institute for Evolutionary Life Sciences (GELIFES), University of Groningen, Groningen, the Netherlands, and University Center of Psychiatry & Interdisciplinary Center of Psychopathology of Emotion Regulation, University Medical Center Groningen, University of Groningen, Groningen, the Netherlands. Email: u.l.m.eisel@rug.nl

A 1. Supplementary methods

Mice

Mice had *ad libitum* access to water and food, and nesting material and a cardboard tube were always present in their cage. Mice were on a 12:12 L/D cycle (lights on at 06:00), in a room with a temperature of 21±2 °C and a humidity of 50%. All mice were housed in the same housing room from 5 months before the start of experiments, and were housed individually starting from approximately 3 months prior to behavioral testing. Behavioral experiments started when mice were 11.5 months of age. All mouse experiments performed in this study were approved by the animal ethics committee of the University of Groningen (DEC6851A).

J20 mice were hemizygous for the J20 transgene, established by standardly crossing J20 mice with WT mice, and Lcn2 KO mice were homozygous for the Lcn2 knock-out. Similarly, J20xLcn2 KO mice were hemizygous for the J20 transgene and homozygous for the Lcn2 knock-out. WT mice in this study were the non-transgenic littermates of the hemizygous J20 transgenic mice. Genotypes of the mice were determined by polymerase chain reaction (PCR) on genomic DNA isolated from tail or ear. The used primers are listed in Supplementary Table 1.

Table S1. Primers used for mouse genotyping.

Target	Forward	Reverse
J20 internal control	5'- CAA ATG TTG CTT GTC TGG TG-3'	5’- GTC AGT CGA GTG CAC AGT TT-3'
J20 transgene	5'- GGT GAG TTT GTA AGT GAT GCC-3'	5’- TCT TCT TCT TCC ACC TCA GC-3'

Lcn2 wildtype	5'- GGT TTG GTG GCA GGC TAT TA-3'	5’- CAG AGT GGC TTT CCC CAT AA-3'
Lcn2 knock-out	5'- TGC TCC TGC CGA GAA AGT AT-3'	5’- CTC TTC CTC CTC CAG CAC AC-3'

Behavioral experiments

To study different behaviors and cognitive functions, several behavioral tests were performed, in the following order: home-cage activity measurement, open field, novel location recognition, Y-maze spontaneous alternation, elevated-plus maze and Morris water maze. All behavioral experiments (except home-cage activity recording) were performed in a separate experimental room, located next to the room in which the mice were housed. All tests (except home-cage activity recording) were filmed by a camera hanging centered above the test arena. For all behavioral tests, the researcher left the experimental room immediately after the start of a test trial. All tests were conducted by the same experimenter, who was blinded for the mouse genotypes. The arenas used for the behavioral tests (except the Morris water maze) were cleaned with ethanol in between mice. In total, 61 mice were studied, with n=13-17 mice per genotype (WT: n=17, J20: n=13, Lcn2 KO: n=16, J20xLcn2 KO: n=15). Mice were studied in two cohorts of 30-31 mice, balanced for genotype.

Habituation

Before start of further behavioral tests, mice were habituated to handling by the researcher, and to the experimental room. The habituation period consisted of 4 days. Each day, mice were handled by the researcher for 2 minutes, in the experimental room. In addition, on the last 2 days of the habituation period, mice were habituated to the arena that would subsequently be used for the open field and novel location recognition tests. For this arena

habituation, mice were placed into the arena for 5 minutes per day. The arena was cleaned with 30% ethanol (leaving behind no excess moisture) between mice. Arena habituation also took place in the experimental room, consistent with the behavioral tests that followed.

Open field

For the open field test, mice were allowed to freely explore a square arena (50x50x36 cm grey Plexiglas floor and walls, no ceiling) for 5 minutes. An overhead camera recorded the mice, and the movements of the mice were tracked with specialized software (EthoVision XT version 11.5, Noldus, The Netherlands). As a measure of locomotor activity, the total distance moved was analyzed. Also, as indications of anxiety-like behavior, the time spent in the center zone (inner 30x30cm) of the arena and the latency to first enter the center zone were measured. Light intensity in the center of the arena was 10-12 lux. The arena was cleaned with 30% ethanol between mice.

Novel location recognition

The day after the open field, the novel location recognition (NLR) test started. The NLR test is a hippocampus-dependent recognition memory task, and is based on rodent's natural exploratory behavior and preference for novelty [1]. The NLR test consisted of a training and test session, which were 24 hours apart. In the NLR training, mice were again placed in the center of the square arena, in which two identical objects were present (metallic soft drink cans, 5.5 cm in diameter and 14 cm high, extended further with a plastic-sheet cone until 24 cm in height to prevent mice from climbing onto the objects). An intra-maze cue (a black and white checkerboard) was added to one of the walls, to allow mice to spatially orient themselves in the arena. Mice were allowed to freely explore the arena and the two objects for

10 minutes. In the NLR training session, the two objects were placed in parallel to either the top or the bottom wall of the arena, separated 10 cm from the walls of the arena.

Twenty-four hours after the training, the NLR test was performed. In the test setting, one of the two objects was placed at its original (training) location, while the other object was relocated towards the other side of the arena (again distanced 10 cm from the walls), creating a diagonal. The intra-maze cue, serving as a stable spatial mark, remained at the same position. Mice were placed in the center of the arena, and were again allowed to explore the arena and objects for 10 minutes. The NLR training and test sessions were recorded by camera, and locomotor behavior and exploration of the objects was analyzed (object exploration could be reliably tracked by the nose-tracking function in EthoVision XT (Noldus)). Object exploration was scored when the nose of the mouse was within a 2 cm radius from the object. Mice with intact recognition memory are expected to recognize objects that are at a familiar place and therefore to spend more time exploring an object that has been placed in a novel location, while mice with memory impairments may not be able to distinguish familiar and novel object locations. As a measure of recognition memory, the discrimination index was calculated as: *(time spent exploring the (to be) relocated object/total exploration time)*100*. A discrimination index of 50% indicated no preference for one of the objects above the other, while a discrimination index of above 50% indicated a preference for the relocated object. Light intensity in the center of the arena was 10-12 lux. The arena was cleaned with 30% ethanol between mice.

Y-maze spontaneous alternation

As an indication of spatial working memory, mice were studied in the Y-maze spontaneous alternation task [2]. Mice were placed in the center of a white Y-shaped maze (with three

arms of 40 cm long, 8 cm wide, and walls of 20 cm high), and allowed to freely explore all three arms for 10 minutes. Trials were recorded by camera and then scored for arm entries (by a blinded observer), after which the percentage of spontaneous alternation was calculated by: *(total number of triads/total number of arm entries – 2)*100*. Herein, a triad is scored when in three consecutive arm entries all three different arms are visited. Light intensity in the center of the arena was 10 lux. The arena was cleaned with 30% ethanol between mice.

Elevated-plus maze

To further assess anxiety-like behavior, exploratory behavior was studied in the elevated-plus maze (EPM) [3,4]. Mice that are more anxious are likely to be more reluctant to explore open and elevated spaces. The EPM used here was a grey plus-shaped maze, consisting of two opposite open arms (no walls) and two closed arms (enclosed by walls, no ceiling), connected in the middle by an open center space. The arms were 30 cm long and 5 cm wide, the closed arms were equipped with 16 cm high walls, and the open center that connected the four arms was 5x5 cm. Mice were placed in the center of the maze (with their nose pointing towards an open arm), and were allowed to freely explore the maze for 8 minutes. The crossings into open and closed arms, and the time spent in the open, closed and center zones were scored. Light intensity in the center of the maze and on the open arms was 10-12 lux. The maze was cleaned with 30% ethanol in between mice.

Morris water maze (hidden-platform)

The Morris water maze (MWM) was performed to assess hippocampus-dependent spatial learning and memory [5]. The MWM was performed as described by Van Dam et al. [6,7], with some modifications. Briefly, the MWM consisted of a circular maze (135 cm in diameter at water level, and walls of 35 cm high), filled with water (23±1 °C, filled to a water level of

20 cm) that was made opaque with non-toxic white paint (Dulux Roll-it easy). A round platform (15 cm in diameter) was hidden at a fixed position in one of the pool quadrants (the target quadrant), submerged 1.5 cm below the water surface. Several visual extra-maze cues were kept at fixed positions, serving as spatial cues. In the training phase, mice were trained to learn the presence and location of the platform. The training phase comprised of eight consecutive days, with one training block per mouse per day. Each daily training block consisted of 4 trials, in each of which a mouse was placed in the border of the pool (with its nose facing towards the border), after which it was allowed to search for the hidden platform for a maximum of 120 s. If a mouse located the platform by itself, it was allowed to sit on the platform for another 15 s, after which it was returned to its home cage. If a mouse was unable to locate the platform within 120 s, it was gently guided towards the platform by the researcher, and was returned to its home cage after sitting on the platform for 15 s. An inter-trial interval of 15 minutes was used, during which mice were allowed to warm up in their cage, placed underneath a heating lamp. Per training block, four different entry positions were used: each trial started from a different quadrant. The order of entry positions (quadrants) per block was varied semi-randomly. The swimming trajectories (including escape latency and swimming distance) of the mice were tracked with EthoVision XT software (Noldus, The Netherlands).

After eight MWM training days, two probe trials followed, performed 24h and 48h after the last training block. For the probe trial, the platform was removed from the maze. Mice were inserted into the maze in the quadrant opposite to the target quadrant, after which they were allowed to swim freely for 100 s. As a measure of spatial accuracy, the number of crossings through the original platform position was scored, as well as the time spent swimming in the target quadrant and the other quadrants. Light intensity was 30 lux in the center of the maze.

Immunohistochemistry for detection of Aβ, GFAP, Iba1 and Lcn2

A week after the last behavioral test was finished, mice were terminally anesthetized by intraperitoneal injection with sodium pentobarbital and transcardially perfused with saline and 4% paraformaldehyde (PFA). Brains were then post-fixed in PFA for an additional 24h, followed by washing in 0.01 M phosphate buffer, and transfer to 30% sucrose for overnight dehydration. Next, dehydrated brains were frozen and then stored at -80 °C until sectioning. Brains were cut in coronal sections of 20 µm thick on a cryostat, and sections were stored in 0.01 M phosphate buffered saline (PBS) with 0.1% sodium azide at 4 °C until stainings were performed. All stainings were done on free-floating hippocampal sections. For all stainings, all incubation steps took place on a shaker. All incubation steps were at room temperature, except for overnight (or longer) incubation steps, which took place in a cold room (4 °C, on a shaker). For the Aβ staining, sections were washed 3x10 min in 0.01M Tris buffered saline (TBS), followed by incubation with 0.3% hydrogen peroxide (H_2O_2) in TBS for 30 min to block endogenous peroxidases. Sections were then washed again, incubated with pre-incubation medium (0.01M TBS containing 0.1% Triton X-100 and 3% goat serum) for 1h, and incubated at 4 °C with the primary antibody (0.01M TBS containing 0.1% Triton X-100, 3% goat serum and 1:2000 6e10 antibody (mouse anti-$A\beta_{1\text{-}16}$, BioLegend, previously Covance)) for 48h. Next, sections were washed and incubated with the secondary antibody (0.01M TBS containing 1% goat serum and 1:400 Biotin-SP-conjugated goat anti-mouse antibody (#115-065-166, Jackson ImmunoResearch Laboratories)) for 2h. After subsequent washing for 3x15 min in TBS, sections were incubated with avidin-biotin complex (1:500 of both components of the Vectastain ABC Elite Kit (#PK6100, Vector Laboratories)) for 2h, washed again and kept in TBS at 4 °C overnight. Finally, the staining was developed with 3,3'-Diaminobenzidine (DAB) at a concentration of 0.7 mg/ml (SigmaFAST DAB tablets

(Sigma)). The DAB reaction in each cup was activated by adding 0.0033% H_2O_2 (end concentration), and stopped by 3 quick washes in TBS. For the GFAP and Iba1 stainings, staining procedures were comparable to that of the Aβ staining, with a few modifications. For the glial fibrillary acidic protein (GFAP) staining, 3% bovine serum albumin (BSA, instead of serum) was used as blocking agent in the pre-incubation medium and primary antibody mix. Sections were incubated at 4 °C with primary antibody (0.01M TBS containing 0.1% Triton X-100, 3% BSA and 1:10 000 GFAP antibody (mouse anti-GFAP, #G3893, Sigma)) overnight. The same goat anti-mouse secondary antibody was used as in the Aβ staining, but now at 1:500 dilution, in 0.01M TBS without further additions. For the ionized calcium binding adaptor protein 1 (Iba1) staining, phosphate buffered saline (PBS) was used instead of TBS. After the first few standard steps (washing in 0.01M PBS, incubation with 0.3% H_2O_2 and washing again), sections were not incubated with a pre-incubation medium, but immediately incubated at 4 °C with a primary antibody solution (0.01M PBS containing 0.1% Triton X-100, 1% BSA and 1:2500 Iba1 antibody (rabbit anti-Iba1, #019-19741, Wako Chemicals)) for 72h. The secondary antibody mix consisted of 0.01M PBS with 1:500 Biotin-SP-conjugated goat anti-rabbit (#111-065-045, Jackson ImmunoResearch Laboratories). For all stainings, after the DAB step, sections were kept in TBS/PBS overnight at 4 °C. Next, the stained sections were mounted with 1% gelatin onto Menzel Superfrost glass microscope slides (Thermo Scientific) and allowed to dry overnight. Subsequently, slices were dehydrated in a series of ethanol to xylol (2x5 min 100% EtOH, 1x5 min 70% EtOH/30% xylol, 1x5 min 30% EtOH/70% xylol, 3x5 min 100% xylol), and coverslipped using DPX (#06522, Sigma) as mounting medium.

For immunofluorescent co-stainings of Lcn2+NeuN, Lcn2+Iba1 and Lcn2+GFAP, sections were washed in 0.01M PBS, followed by incubation with pre-incubation medium (0.01M

PBS containing 0.05% Triton X-100 and 3% normal donkey serum) for 40 min. Sections were then incubated overnight with the primary antibody mixes (Lcn2 antibody, combined with one of the appropriate other antibodies (NeuN, Iba1 or GFAP antibody)). The antibody mixes were prepared in 0.01M PBS with 3% donkey serum, and used antibody concentrations were 1:1000 for Lcn2 (rat anti-Lcn2, #Ab70287, Abcam), 1:500 for NeuN (mouse anti-NeuN, #MAB377, Chemicon), 1:2000 for Iba1 and 1:10 000 for GFAP. The Iba1 and GFAP antibodies used were the same as the antibodies described above. After overnight incubation, sections were washed and incubated with Alexa Fluor-conjugated secondary antibodies (all from ThermoFisher Scientific) for 2h. The secondary antibodies used were AF488-conjugated donkey anti-rat (#A21208) for detecting Lcn2, AF594-conjugated donkey anti-mouse (#21203) for detecting NeuN and GFAP, and AF555-conjugated donkey anti-rabbit (#A31572) for detecting Iba1. Subsequently, sections were washed in PBS, mounted onto microscope slides in PBS, and coverslipped with Mowiol 4-88 (#81381, Sigma) as mounting medium. Transparent nailpolish was applied at the edges to seal the slide and coverslip. Fluorescent stainings were imaged using the Leica SP8 confocal laser scan microscope. These stainings were performed to obtain a qualitative indication of Lcn2 localization, and were not used for further quantitative analyses.

Histochemistry for detection of iron

For detection of non-heme (mostly ferric Fe^{3+}) iron the DAB-enhanced Perls' iron stain was used, as described previously [8–11]. A staining protocol comparable to that reported by Chen et al., 2015 [8] was used, with some modifications. Briefly, free-floating hippocampal sections were washed in 0.01 M PBS for 3x15 min, after which endogenous peroxidases were blocked by incubation with 0.3% H_2O_2 for 30 min. Subsequently, sections were washed again for 4x15 min in PBS, and Perls' solution (1:1 mix of 5% potassium ferrocyanide and 5% HCl,

both prepared in milliQ H_2O) was prepared shortly before use. Sections were then incubated in Perls' solution for 50 min, and washed in milliQ H_2O for 3x15 min. DAB reaction was performed as described above, however now including Nickel (2.5 mg Nickel ammonium sulfate per 5 ml DAB solution) to further enhance the staining, and using 0.01% H_2O_2 (end concentration) for activation of DAB. The DAB reaction was stopped (in this case after 45 min) by 3 quick washes in PBS. After overnight washing in PBS at 4 °C sections were mounted in 1% gelatin and allowed to dry overnight, followed by dehydration and coverslipping using DPX. Iron staining was performed in the dark as much as possible. As mentioned, the Perls' iron stain mostly stains for Fe^{3+} iron (and some Fe^{2+}). Yet, the detection of Fe2+ may have been enhanced to some extent due to pre-treatment with H_2O_2; besides blocking of endogenous peroxidases, H_2O_2 might be expected to *in situ* oxidize some Fe^{2+} to Fe^{3+} [11].

Quantification of (immuno)histochemical stainings

For the Aβ, GFAP and iron stainings, the coverage as well as the optical density of positively stained structures was quantified. This quantification was done with a macro written in LAS Macro Editor (Leica Microsystems), enabling the drawing of a region of interest in a fixed image, after which the coverage and optical density within the region of interest were automatically measured. Analysis of the Aβ staining was performed at 50x magnification, while analyses of the GFAP and iron stainings were performed at 100x magnification for sufficient detail in astrocytic processes and iron depositions (all analyses were done using the Leica DMI6000 B microscope, Leica microsystems, Rijswijk, The Netherlands). For analysis of the Iba1 staining, we were interested in obtaining a measure of microglial activation. As described previously by Hovens et al. [12,13], the ratio between the cell body size and the total cell size of microglia may give an indication of microglial activation, since microglia

activation has in general been related to a morphological transition in which microglia retract their processes and gain in cell body size. To this end, pictures of the Iba1 staining were made at 200x magnification (Olympus BH2 microscope, using Leica QWin software) to have sufficient detail in microglial processes, after which pictures were further analyzed with Image-Pro Plus 6.0 (Media Cybernetics, Rockville, MD, USA). Firstly, a region of interest was drawn in which all positively stained pixels were counted (using the 'automatic dark objects' function), representing staining of total cells within the region of interest. Next, only the pixels representing the cell bodies in the same region of interest were measured, which required setting of a size (in this study: 400-10 000 000 pixels) and intensity range (in this study: 0-140 pixel values). With the obtained values, an indication of microglial activity was calculated as follows: *(cell body size/total cell size)*100*. Moreover, the number of microglia per area of interest and coverage of Iba1-positive staining were measured. For all stainings, quantification was mostly focused on the hippocampus. The Aβ staining was analyzed for the entire hippocampus. The analysis of the GFAP, Iba1 and iron stainings was performed for specific hippocampal sub-regions, including the stratum oriens and stratum radiatum of the cornu ammonis sector 1 (CA1) region, and the inner and outer blade of the dentate gyrus (DG). For the iron staining also the pyramidal and granular layers of the CA1 and DG were included, as well as the stratum lacunosum moleculare (analyzed hippocampal sub-regions are also indicated in Supplementary Fig.1). Of note: the (intracellular) iron staining in the CA1 pyramidal and DG granular cell layers was less intense (yet clearly present), as compared to the intense (plaque-associated) iron staining in the other studied hippocampal sub-regions. Therefore, proper detection of the iron staining in the CA1 pyramidal and DG granular cell layers required an adjusted detection threshold during analysis, which explains the higher relative coverages of iron staining found for these regions (see Figure 4). For all stainings, 3-6 hippocampi were analyzed per mouse.

A 2. Supplementary results

Figure S1

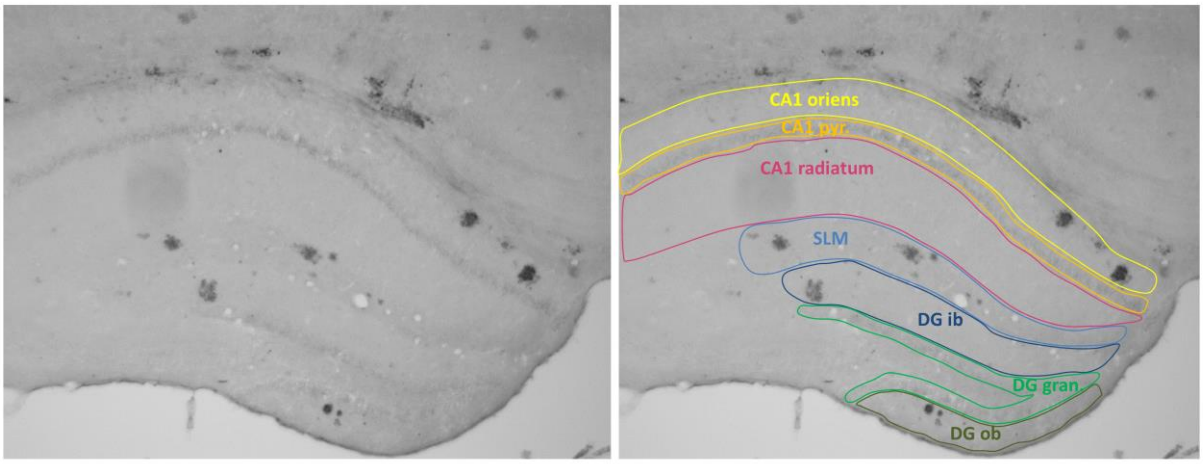

Figure S1

Illustration of hippocampal sub-regions that were analyzed for the Perls' iron staining. Analyzed hippocampal sub-regions include the CA1 oriens, CA1 pyramidal layer (CA1 pyr), CA1 radiatum, stratum lacunosum moleculare (SLM), Dentate gyrus inner blade (DG ib), DG granular layer (DG gran) and DG outer blade (DG ob). Analyzed hippocampal sub-regions are indicated in a picture at 50x magnification, to allow for indication of the entire analyzed sub-regions. However, the real analyses were performed at 100x magnification; therefore, the CA1 sub-regions and SLM were analyzed in 2 halves, to measure the entire sub-regions.

Figure S2

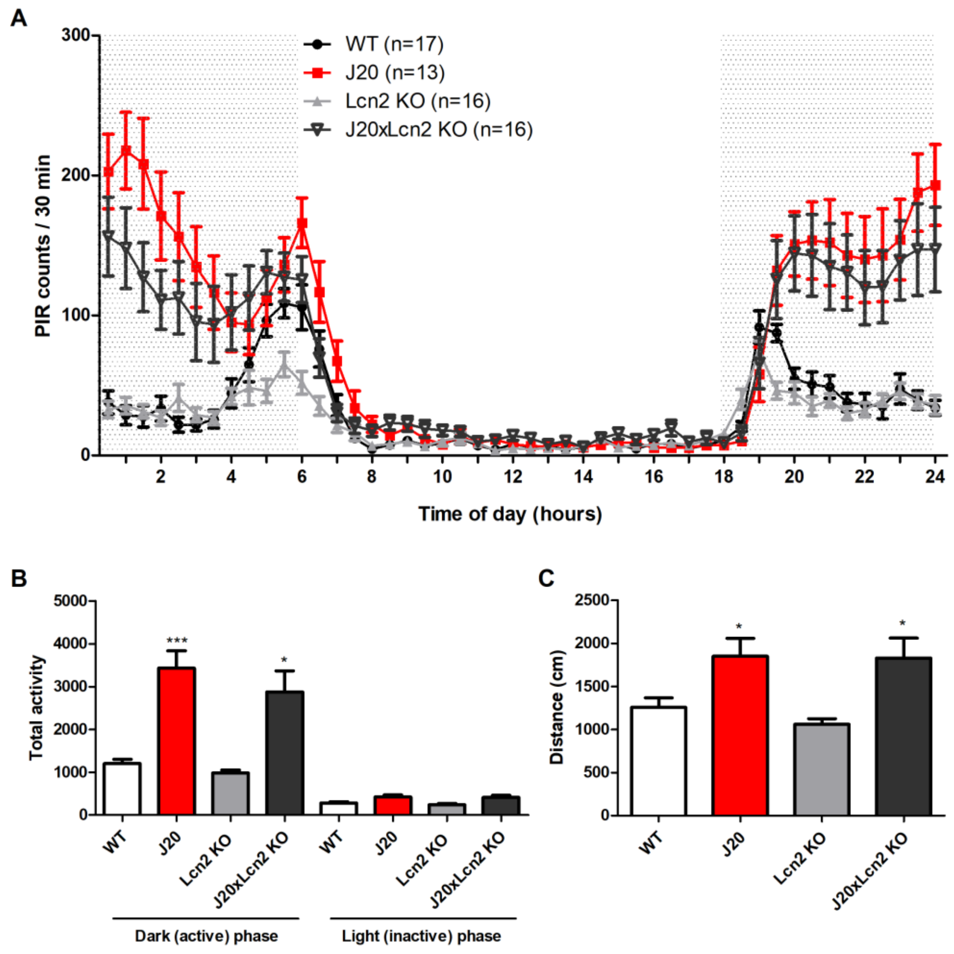

Figure S2

J20 and J20xLcn2 KO mice present hyperactive behavior. (A) Average daily pattern of home-cage activity, shown as the average activity per 30 minutes. Grey background shading indicates the dark (active) phase (from 18:00 until 06:00). (B) Total daily activity analyzed for the dark and light phase. Tested with Kruskal-Wallis test with Dunn's multiple comparisons post-hoc test. (C) The distance traveled in the open field (5 minutes). Tested with one-way ANOVA with Tukey's multiple comparisons test. n= 13-17 mice per group (WT n=17, J20 n=13, Lcn2 KO n=16, J20xLcn2 KO n=15 in the open field and n=16 in the home-cage activity measurement). * $p < 0.05$ and *** $p < 0.0001$ compared to WT. No significant difference was present between J20 and J20xLcn2 KO mice.

Figure S3

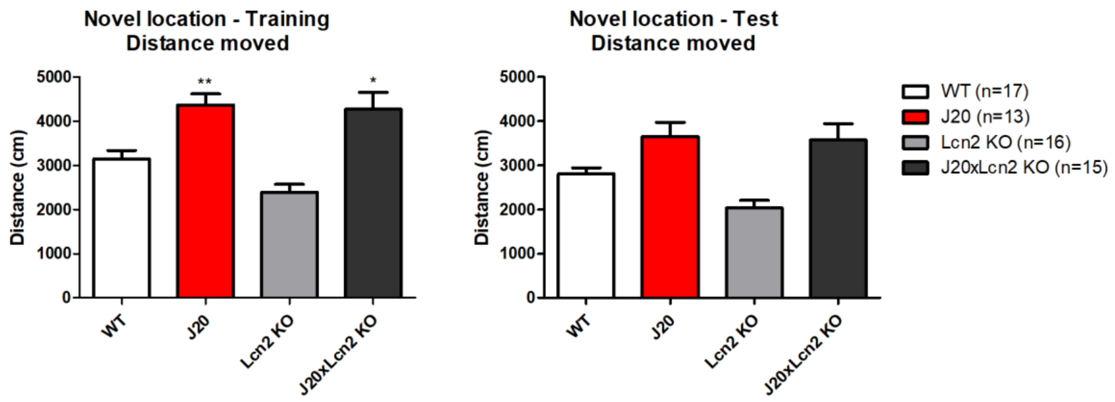

Figure S3

Hyperactive locomotor behavior is also visible in the NLR training (left) as indicated by increased distance moved. A tendency towards hyperactive locomotor behavior remains present in the NLR test (right). Tested with one-way ANOVA with Tukey's multiple comparisons test. n= 13-17 mice per group (WT n=17, J20 n=13, Lcn2 KO n=16, J20xLcn2 KO n=14). * $p < 0.05$ and ** $p < 0.01$ compared to WT.

Figure S4

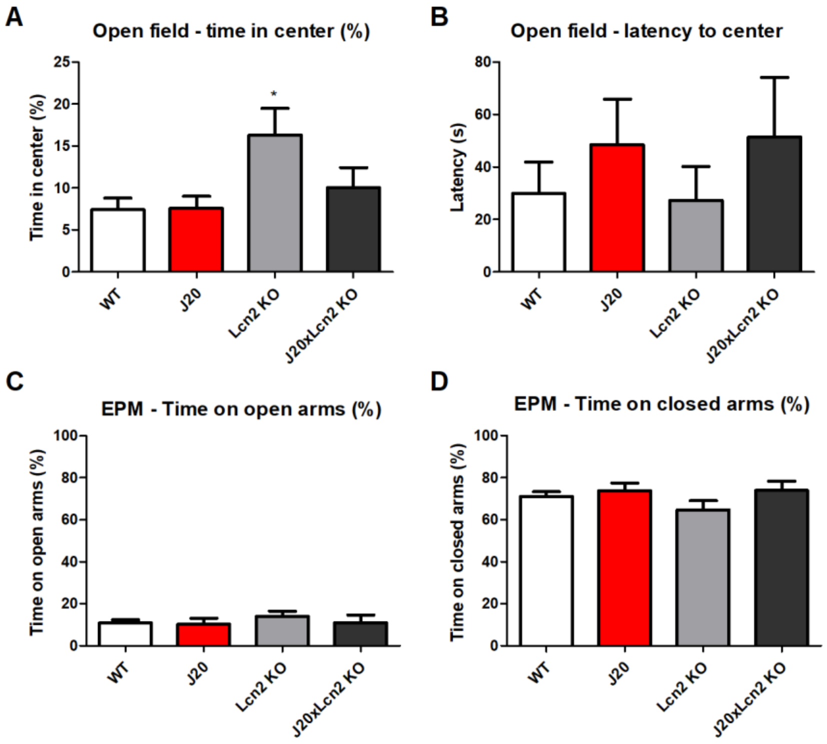

Figure S4

No clear differences in anxiety-like behavior between genotypes. (A) Time spent in the center of the open field (% of total time, 5 minutes). (B) Latency (s) to enter the center of the open field. (C) Time spent on the open arms of the elevated-plus maze (EPM) (% of total time, 8 minutes). (D) Time spent on the closed arms of the EPM (% of total time, 8 minutes). Tested with one-way ANOVA with Tukey's multiple comparisons test. n= 13-17 mice per group (WT n=17, J20 n=13, Lcn2 KO n=16, J20xLcn2 KO n=15). * $p < 0.05$.

Figure S5

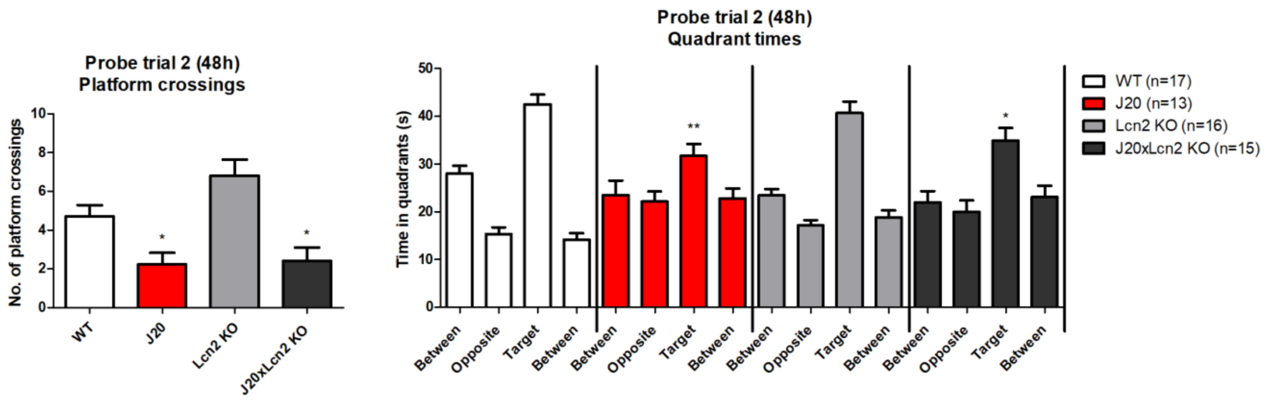

Figure S5

Results of the second MWM Probe trial, performed 48h after the last training trial. The results of this second probe trial are comparable to the results of the first probe trial, which was performed 24h after the last training. Tested with one-way ANOVA followed by Dunnett's multiple comparisons test. n= 13-17 mice per group (WT n=17, J20 n=13, Lcn2 KO n=16, J20xLcn2 KO n=15). * $p < 0.05$ and ** $p < 0.01$, compared to WT.

Figure S6

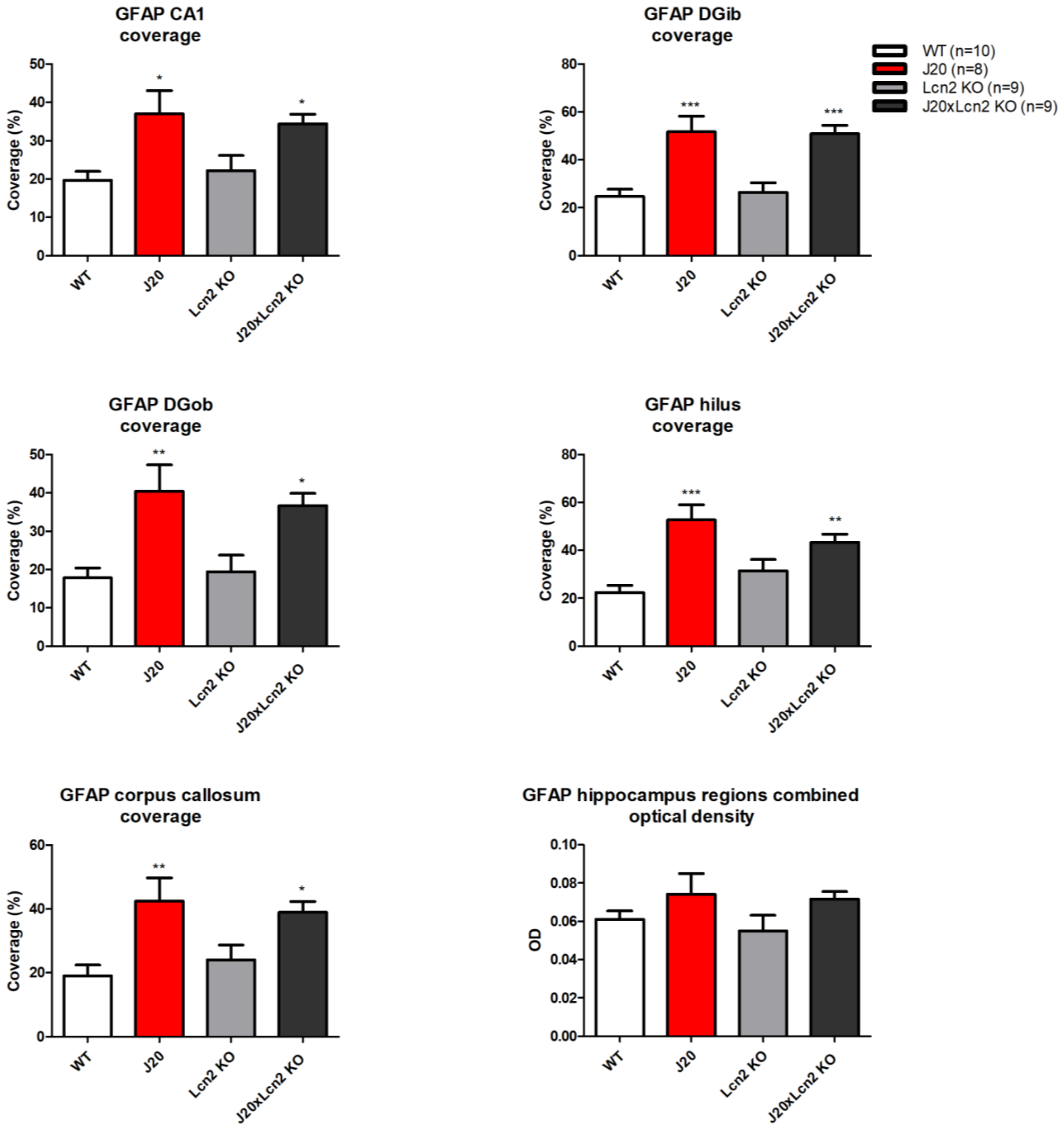

Figure S6

Additional measurements of the GFAP staining, including the coverage of the GFAP staining in specific hippocampal regions: CA1, dentate gyrus inner blade (DG ib), dentate gyrus outer blade (DG ob) and the hilus. In addition, the coverage of GFAP staining was analyzed in the corpus callosum. Also, the optical density (OD) of the GFAP staining in the hippocampus

(combined CA1, GD and hilus measurements) was analyzed. n= 9-10 mice per group (WT n=10, J20 n=8, Lcn2 KO n=9, J20xLcn2 KO n=9), 3-6 hippocampi were analyzed per mouse. Tested with one-way ANOVA with Tukey's multiple comparisons test. * $p < 0.05$, ** $p < 0.01$ and *** $p < 0.0001$ compared to WT. No significant differences were present between J20 and J20xLcn2 KO mice.

Figure S7

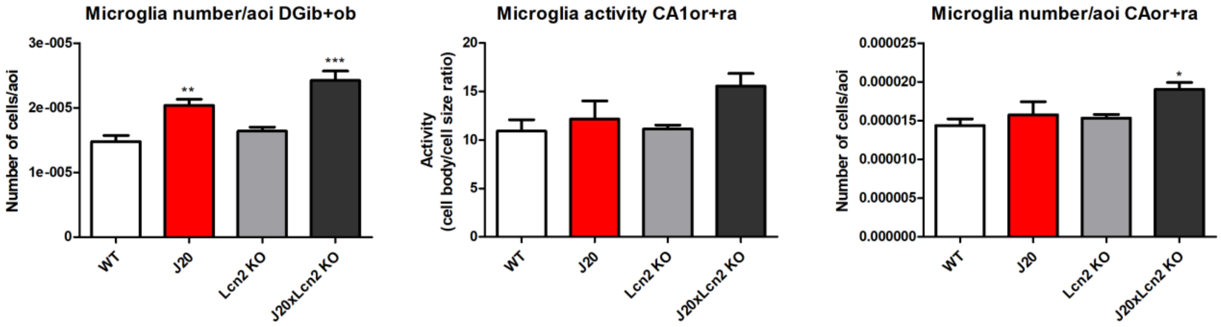

Figure S7

Additional measurements of the Iba1 microglia staining, including the number of microglia counted (per area of interest) in the dentate gyrus (DG inner blade and outer blade combined). In the middle and right figures the microglia activity and number of microglia (per area of interest) in the CA1 hippocampal region (CA1 stratum oriens and stratum radiatum combined) are depicted. n= 9-10 mice per group (WT n=10, J20 n=8, Lcn2 KO n=9, J20xLcn2 KO n=9), 3-6 hippocampi were analyzed per mouse. Tested with one-way ANOVA with Tukey's multiple comparisons test. * $p < 0.05$, ** $p < 0.01$ and *** $p < 0.0001$ compared to WT.

Figure S8

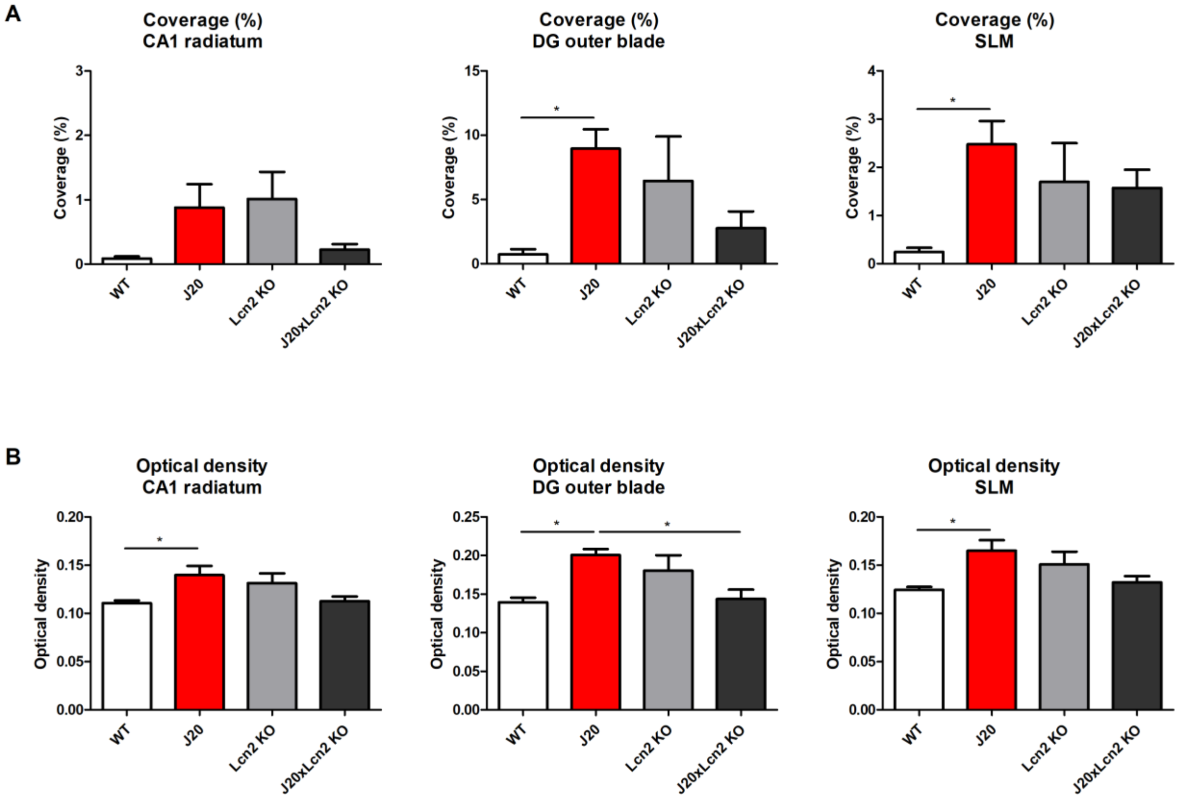

Figure S8

Additional measurements of the Perls' iron staining, including the coverage and optical density of iron staining in the CA1 radiatum, Dentate gyrus outer blade (DG outer blade), and stratum lacunosum moleculare (SLM). (A) Quantification of the coverage of iron-positive staining, in the CA1 radiatum, DG outer blade and SLM. (B) Quantification of the optical density of iron-positive staining, in the CA1 radiatum, DG outer blade and SLM. Images were taken and analysis was performed at 100x magnification. n= 8-9 mice per group (WT n=9, J20 n=8, Lcn2 KO n=9, J20xLcn2 KO n=9), 3-4 hippocampi were analyzed per mouse. Tested with Kruskal-Wallis test with Dunn's multiple comparisons post-hoc test. * $p < 0.05$.

Figure S9

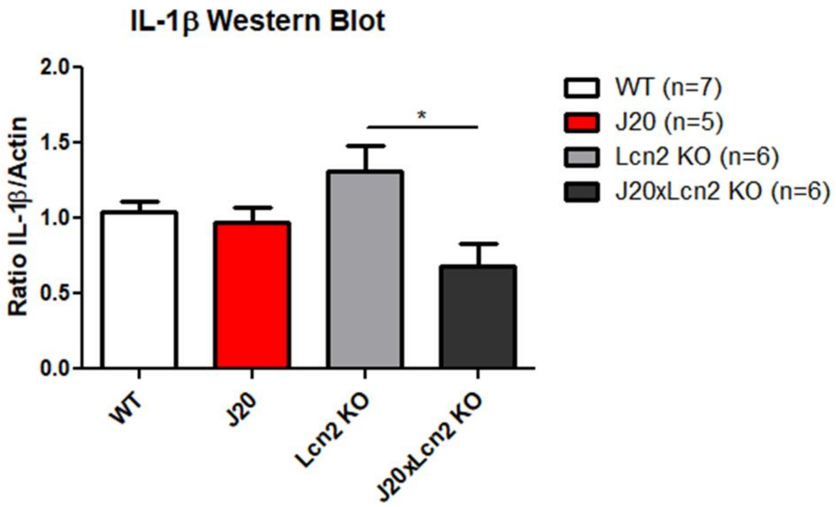

Figure S9

Western blot analysis of IL-1β levels in hippocampal lysates of WT (n=7), J20 (n=5), Lcn2 KO (n=6) and J20xLcn2 KO (n=6) mice. Three technical repeats were performed for all samples. IL-1β levels were corrected for actin, which served as internal control to control for loading variations. Primary antibody used for detection of IL-1β is ab9722 (Abcam). Tested with one-way ANOVA with Tukey's multiple comparisons test. * $p < 0.05$.

Figure S10

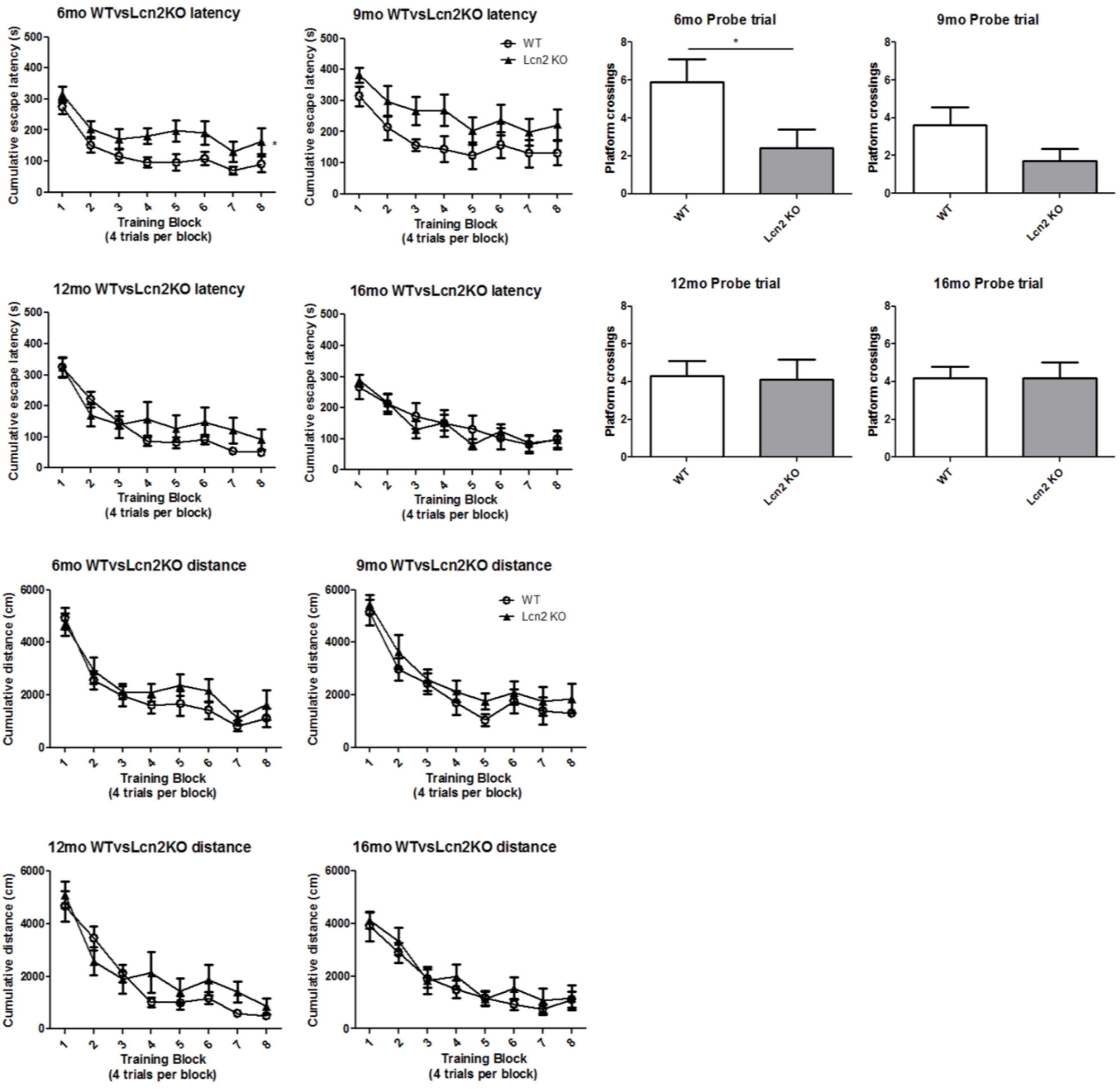

Figure S10

MWM performance of WT and Lcn2 KO mice of 6, 9, 12 and 16 months of age. The top four curve graphs show the escape latency (s), and the bottom four curve graphs show the swim distance (cm) during the MWM training; tested with two-way repeated measures ANOVA. The four bar graphs on the right show the number of platform crossings in the probe trial, 24h

after the last training; tested with unpaired two-tailed t test. n=10 mice per group. * $p < 0.05$ compared to WT.

A 3. Supplementary references

1. Vogel-Ciernia A, Wood MA. Examining Object Location and Object Recognition Memory in Mice. Curr Protoc Neurosci Editor Board Jacqueline N Crawley Al. 2014;69:8.31.1-8.31.17.

2. Wolf A, Bauer B, Abner EL, Ashkenazy-Frolinger T, Hartz AMS. A Comprehensive Behavioral Test Battery to Assess Learning and Memory in 129S6/Tg2576 Mice. PloS One. 2016;11:e0147733.

3. Rodgers RJ, Dalvi A. Anxiety, defence and the elevated plus-maze. Neurosci Biobehav Rev. 1997;21:801–10.

4. Walf AA, Frye CA. The use of the elevated plus maze as an assay of anxiety-related behavior in rodents. Nat Protoc. 2007;2:322–8.

5. D'Hooge R, De Deyn PP. Applications of the Morris water maze in the study of learning and memory. Brain Res Brain Res Rev. 2001;36:60–90.

6. Van Dam D, Coen K, De Deyn PP. Cognitive evaluation of disease-modifying efficacy of donepezil in the APP23 mouse model for Alzheimer's disease. Psychopharmacology (Berl). 2008;197:37–43.

7. Van Dam D, Lenders G, De Deyn PP. Effect of Morris water maze diameter on visual-spatial learning in different mouse strains. Neurobiol Learn Mem. 2006;85:164–72.

8. Chen Q, Tang J, Tan L, Guo J, Tao Y, Li L, et al. Intracerebral Hematoma Contributes to Hydrocephalus After Intraventricular Hemorrhage via Aggravating Iron Accumulation. Stroke. 2015;46:2902–8.

9. Meguro R, Asano Y, Odagiri S, Li C, Iwatsuki H, Shoumura K. Nonheme-iron histochemistry for light and electron microscopy: a historical, theoretical and technical review. Arch Histol Cytol. 2007;70:1–19.

10. van Duijn S, Bulk M, van Duinen SG, Nabuurs RJA, van Buchem MA, van der Weerd L, et al. Cortical Iron Reflects Severity of Alzheimer's Disease. J Alzheimers Dis JAD. 2017;60:1533–45.

11. Smith MA, Harris PLR, Sayre LM, Perry G. Iron accumulation in Alzheimer disease is a source of redox-generated free radicals. Proc Natl Acad Sci U S A. 1997;94:9866–8.

12. Hovens IB, van Leeuwen BL, Nyakas C, Heineman E, van der Zee EA, Schoemaker RG. Postoperative cognitive dysfunction and microglial activation in associated brain regions in old rats. Neurobiol Learn Mem. 2015;118:74–9.

13. Hovens IB, Nyakas C, Schoemaker RG. A novel method for evaluating microglial activation using ionized calcium-binding adaptor protein-1 staining: cell body to cell size ratio. Neuroimmunol Neuroinflammation. 2014;82–8.
